# Health-related quality of life and mental health in autoimmune thrombotic thrombocytopenic purpura patients in the caplacizumab era

**DOI:** 10.1016/j.rpth.2025.103297

**Published:** 2025-12-09

**Authors:** Julia Weisinger, Christina Tites, Laurent Gilardin, Aki Baba, Ygal Benhamou, Elie Azoulay, Jean-Francois Augusto, Thomas Papo, Claire Cartery, Olivier Moranne, Gabriel Choukroun, Loïc Lièvre, Jehane Fadlallah, Pascale Poullin, Arnaud Jaccard, Claire Presne, Bérangère Joly, Agnès Veyradier, Raida Bouzid, Paul Coppo

**Affiliations:** 1 Centre de Référence des Microangiopathies Thrombotiques, Service d'hématologie, Hôpital Saint Antoine, APHP and Sorbonne Université (AP-HP.6), Paris, France; 2 Service de Médecine interne, Hôpital Jean Verdier, AP-HP. Hôpitaux Universitaires Paris Seine-Saint-Denis, Bondy, France; 3 Service de Médecine interne, Hôpital Saint Antoine, AP-HP. Sorbonne Université, Paris, France; 4 Service de Médecine Interne, CHU Charles Nicolle, Rouen, France; 5 Médecine Intensive Réanimation, Hopital Saint Louis, APHP, Paris, France; 6 Service de Néphrologie-Dialyse-Transplantation, CHU d’Angers, Angers, France; 7 Service de Médecine Interne, Hopital Bichat, APHP, Paris, France; 8 Service de Néphrologie, CH de Valenciennes, Valenciennes, France; 9 Service de Néphrologie, Dialyses Apherese, Hopital Universitaire de Nimes, IDESP, Université de Montpellier, France; 10 Service de néphrologie et de transplantation, CHU d'Amiens, Amiens, France; 11 Service de Néphrologie, Hopital Maison Blanche, Reims, France; 12 Service d'Immunologie Clinique, Hôpital Saint-Louis, APHP, Paris, France; 13 Service d'Hémaphérèse, Hôpital de La Conception, CHU de Marseille, Marseille, France; 14 Service d’Hématologie et de Thérapie Cellulaire, CHU Limoges, Limoges, France; 15 Department of Adult Nephrology, CHU d'Amiens, Amiens, France; 16 INSERM Unité Mixte de Recherche (UMRS) 1138, Centre de Recherche des Cordeliers, Paris, France; 17 Service d'Hématologie biologique, Hôpital Lariboisière, AP-HP.Nord, Université Paris Cité, Paris, France

**Keywords:** caplacizumab, mental disorders, neurologic disturbances, quality of life, TTP

## Abstract

**Background:**

Despite improvement in acute care of immune-mediated thrombotic thrombocytopenic purpura (iTTP), numerous studies showed that patients with iTTP have inferior mental health and health-related quality of life (HRQoL). Caplacizumab led to shorter hospitalization, less plasma exchange, and improved survival in iTTP and might influence long-term HRQoL.

**Objectives:**

We aimed to address the impact of iTTP on HRQoL, posttraumatic stress disease, depression, and anxiety, as well as the possible role of caplacizumab on improving these features.

**Methods:**

We conducted a survey among patients with iTTP enrolled in the French thrombotic microangiopathy registry: patients completed the Short Form (SF)-36 HRQoL, the Hospital Anxiety and Depression Scale screening for anxiety and depression, and the Posttraumatic Stress Disorder Checklist for DSM-IV posttraumatic stress disease questionnaires. Results were compared to those of a sample of the French general population.

**Results:**

A total of 101 patients with iTTP in remission (45 patients previously treated with caplacizumab and 56 patients without caplacizumab) and 76 healthy controls were included. Patients with iTTP had significantly lower scores in all domains in the SF-36 survey and higher anxiety and depression scores than healthy controls. Advanced age was associated with improved SF-36 scores, lower anxiety scores and less severe anxiety cases. Furthermore, the use of caplacizumab led to a lower risk of severe anxiety.

**Conclusion:**

Based on our results, HRQoL is decreased in patients with iTTP, and depression and anxiety are more prevalent. Caplacizumab treatment might influence long-term mental and psychological outcomes in iTTP by shortening the duration of treatment with plasma exchange.

## Essentials

• Patients with iTTP have inferior mental health and HRQoL.
• We assessed HRQoL, depression, anxiety and PTSD in patients with iTTP.
• Patients with iTTP have decreased HRQoL and higher anxiety and depression scores.
• Caplacizumab was associated with less severe anxiety and might influence quality of life.

## Visual Abstract

**Figure:**
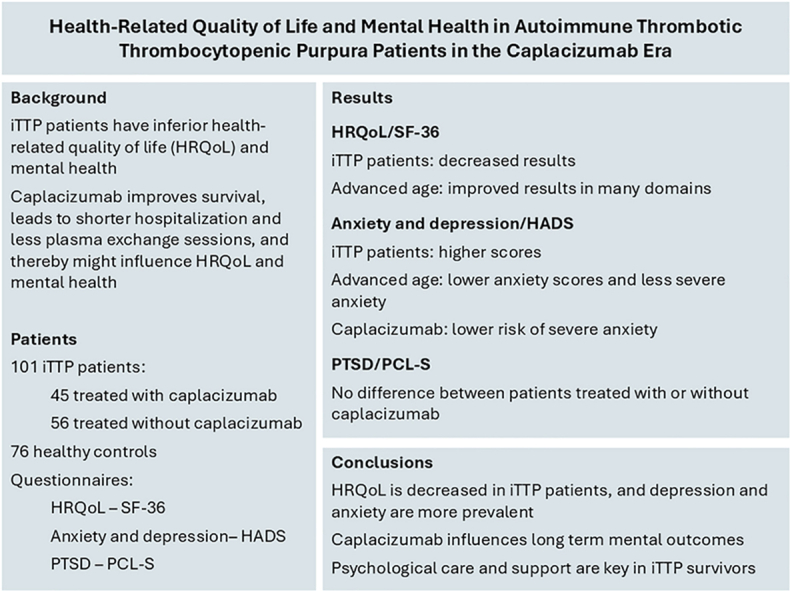

## 1 Introduction

Immune-mediated thrombotic thrombocytopenic purpura (iTTP) is a rare life-threatening disease caused by an immune, antibody-mediated, deficiency of the von Willebrand factor cleaving protease ADAMTS-13 (a disintegrin and metalloproteinase with thrombospondin-1 motifs, 13th member), leading to widespread microthrombi [1,2]. If left untreated, this condition is fatal in >90% of cases. Standard treatment consists in therapeutic plasma exchange (TPE), immunosuppression with glucocorticoids and the B cell–depleting monoclonal antibody rituximab, and targeting von Willebrand factor with the nanobody caplacizumab. With this regimen, the outcome of the acute phase improved substantially in the recent years, with survival rates of >95% [3]. However, the high burden of neurologic and mental health comorbidities in those patients has an important impact on patients’ health-related quality of life (HRQoL) [4,5]. Many studies proved that HRQoL is inferior in patients with iTTP than that in the normal population, after the acute phase of TTP; furthermore, depression, anxiety, and posttraumatic stress disorder (PTSD) are more common [[4], [5], [6], [7]]. In addition, HRQoL does not seem to improve with time from acute episode [4,5]. In the last decades, new therapies such as rituximab and, more recently, caplacizumab emerged and significantly improved both survival and the burden of care in iTTP. In particular, caplacizumab leads to a shorter hospitalization stay, especially in the intensive care unit; therefore, it might have a beneficial role on HRQoL [3,8]. So far, the possible effect of caplacizumab on long-term mental health and HRQoL only involved studies with a limited number of patients [9] and therefore deserves further investigation. Here, we addressed the impact of iTTP on HRQoL, PTSD, depression, and anxiety, as well as the possible role of caplacizumab on improving these features.

## 2 Methods

### 2.1 Patients and recruitment

We assessed HRQoL, anxiety, depression, and PTSD in patients with iTTP and compared these findings to those of a healthy control group. The study was conducted using questionnaires submitted to patients with iTTP recruited in the Registry of the French National Reference Center for Thrombotic Microangiopathies (CNR-MAT, www.cnr-mat.fr). We included adult (≥18 years old) patients with a previous diagnosis of iTTP as defined by a severe, antibody-mediated ADAMTS-13 deficiency, in clinical remission at time of inclusion [10]. The recruitment period ranged from December 2007 and July 2020. Patients had to be able to read and understand French. Data on patients with a diagnosis of iTTP were collected according to a predefined computerized dataset [11,12]. Diagnostic criteria, remission, and relapse definitions were based on previous studies [10,13,14].

### 2.2 Management

Treatment of iTTP in the acute phase was based on current national and international guidelines [13,15]. Briefly, acute phase treatment consisted of daily TPE started at diagnosis and carried out until clinical response. Patients received glucocorticoids (1 mg/kg/d and then tapered, for a total of 3 weeks). Rituximab has been routinely used since 2007, first as salvage therapy and then frontline when caplacizumab was approved for the treatment of iTTP; caplacizumab has been used routinely since September 2018 [8,15,16]. In the pre-emptive setting, rituximab was started after detection of a severe ADAMTS-13 deficiency (activity < 20%) [11,12].

### 2.3 Questionnaires

The survey included 4 questionnaires. The first included general questions about previous episodes of anxiety or depression, previous health care consultation or medication for anxiety or depression, employment status before and after iTTP diagnosis, and difficulties for returning to work. The second questionnaire consisted of the Short Form (SF)-36 questionnaire that assesses HRQoL; it contains 36 items evaluating 8 domains: physical functioning, role limitations due to physical problems, bodily pain, general health perceptions, vitality, social functioning, role limitations due to emotional problems, and mental health. Scores range from 0 to 100 for each of the 8 domains and higher scores indicate better quality of life [17,18]. The physical composite score (PCS) and mental composite score (MCS) are calculated according to established country-specific algorithms to have the same mean and SD in each country (50 and 10). The raw scores were converted to a 0 to 100 scale and transformed to standardized *z*-scores for calculating the summary scores of MCS and PCS. The third questionnaire used was the Hospital Anxiety and Depression Scale (HADS), which can be used as a screening tool for anxiety and depression with a recall time of 1 week. It can be used in the hospital or the outpatient setting. The HADS has 2 scales with 7 items for anxiety and 7 for depression, with a total score range from 0 to 21 each. High screening scores indicate more severe symptoms, with scores from 15 to 21 classifying as severe, 11 to 14 moderate, 8 to 10 mild, and 0 to 7 being normal [19]. The last questionnaire was the French version of the Posttraumatic Stress Disorder Checklist for DSM-IV (PCL-S). PCL-S applies to describe reactions to a specific event that must be identified by the respondent [20]. The specific event in our study was linked to the patient’s iTTP diagnosis and hospitalization; therefore, this questionnaire was only completed by patients with iTTP and not by the healthy controls. Participants received the questionnaires through mail or during their iTTP follow-up clinical visits. The healthy controls were recruited among the investigators’ entourage and had to be 18 years or older, able to read, and understand French.

### 2.4 Statistical analysis

Data were summarized as counts and percentages. Medians and lowest and highest values or IQR were indicated. To compare variables, Fisher exact test or Mann–Whitney U-test were used. To assess risk factors, linear, robust linear, logistic and β regression models were performed, as applicable. Statistical power analysis and sample size calculation were performed (Supplementary Table 1). Analyses were performed using IBM SPSS statistical software (IBM SPSS Statistics for Windows; version 22.0; IBM Corp) and RStudio and R software 4.5.0 (R Foundation). Statistical significance was set at *P* < .05.

### 2.5 Ethics

This study was part of the Thrombotic Microangiopathy program study approved by the Ethics Committee of Hospital Pitié-Salpêtrière (Paris, France; www.clinicaltrials.gov, NCT00426686), the Health Authority and the French Ministry of Health (P051064/PHRC AOM05012), and the French Data Protection Authority. Study procedures were performed in accordance with the Declaration of Helsinki.

## 3 Results

### 3.1 Patients and baseline characteristics

**Figure 1:**
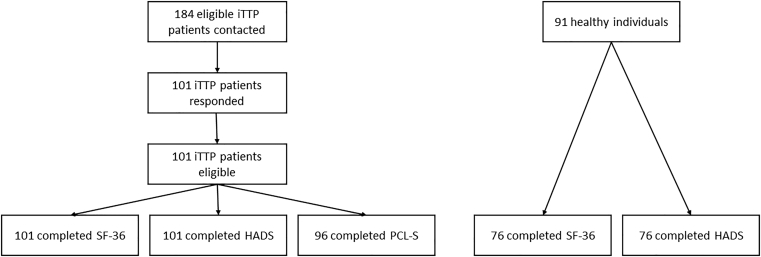
Study flow chart. HADS, Hospital Anxiety and Depression Scale; iTTP, immune-mediated thrombotic thrombocytopenic purpura; PCL-S, Posttraumatic Stress Disorder Checklist for DSM-IV; SF-36, Short form-36.

In total, 184 eligible patients with iTTP were contacted between January and August 2021; among them, 101 patients (55%) from 21 centers responded to the survey and were analyzed in this study. All of them completed both the SF-36 and the HADS questionnaires, while 96 of them completed the PCL-S survey. Forty-five patients (44.5%) received caplacizumab. Ninety-one healthy controls were contacted; 76 completed the SF-36 and the HADS questionnaires (flow chart of the study detailed in Figure 1). Median time from iTTP diagnosis to the survey was 47 months (IQR, 29-84 months).

Baseline characteristics of patients with iTTP are detailed in Table 1. Significant differences were detected: in caplacizumab-treated patients, lactate dehydrogenase levels were higher (*P* = .03), the number of TPE was lower (*P* < .001), and rituximab use was more common (*P* = .009) than those in patients treated without caplacizumab [15]. As expected, follow-up was shorter in caplacizumab-treated patients (*P* < .001). The number of patients hospitalized in intensive care unit, as well as hospitalization in intensive care unit, was comparable between patients treated with and those without caplacizumab. No significant difference was detected regarding age, sex (ie, male or female), and self-reported previous anxiety and depression between patients with iTTP and healthy controls and between patients treated with iTTP and those without caplacizumab (Supplementary Table 2; Table 1).

**Table 1:** Characteristics of patients with iTTP.

Characteristic	All iTTP (*N* = 101)	No caplacizumab (*n* = 56)	Caplacizumab (*n* = 45)	*P*
Age (y)	51 (38-62)	51 (39-58)	51 (37-65)	.98
Female	76 (75)	44 (79)	32 (71)	.49
Previous health care visit due to mood disorder or stress	31 (31)	14 (25)	17 (38)	.28
Previous medication due to mood disorder or insomnia	15 (15)	6 (11)	9 (20)	.26
Acute phase				
CNS involvement	65 (64)	35 (63)	30 (67)	.68
Cardiac involvement	41 (41)	19 (34)	22 (49)	.15
LDH, × ULN	4.48 (2.96-6.00)	4.12 (2.47-5.63)	5.24 (3.70-6.22)	.03
Platelet count (G/L)	14 (10-20)	13 (8-19)	16 (10-23)	.41
Hemoglobin (g/dL)	8.6 (7.4-9.8)	8.5 (7-9.9)	8.8 (7.7-9.8)	.22
Serum creatinine (μmol/L)	85 (70-117)	81 (69-114)	92 (70-120)	.36
TPE treatment	101 (100)	56 (100)	45 (100)	>.999
No. of TPE per patients	6 (5-14)	12 (5-18)	5 (4-6)	<.001
Corticosteroid treatment	95 (94)	53 (95)	42 (93)	>.999
Rituximab treatment	76 (75)	36 (64)	40 (89)	.009
Hospitalization on ICU	72 (71)	37 (66)	35 (78)	.27
Days per patient on ICU	5 (3-9)	6 (3-12)	5 (3-8)	.47
Follow-up				
Time from diagnosis to survey (mo)	47 (29-84)	71 (51-119)	29 (25-34)	<.001
Clinical relapse	19 (19)	11 (20)	8 (18)	>.999
ADAMTS-13 relapse	33 (33)	22 (39)	11 (24)	.200

Values are median (IQR) or *n* (%).

ADAMTS, A disintegrin and metalloproteinase with thrombospondin-1 motifs; CNS, central nervous system; ICU, intensive care unit; iTTP, immune-mediated thrombotic thrombocytopenic purpura; LDH, lactate dehydrogenase; TPE, therapeutic plasma exchange; ULN, upper limit of normal.

### 3.2 Health-related quality of life

**Figure 2:**
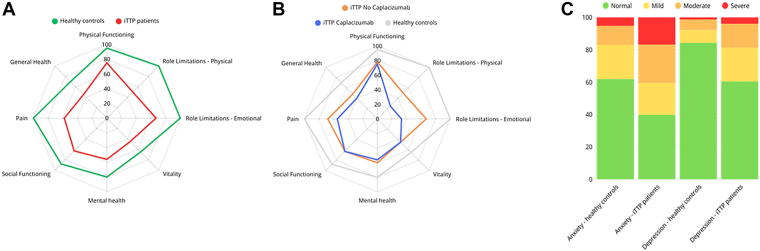
Results of the SF-36 and the HADS surveys. (A) Comparison of SF-36 results of patients with iTTP and healthy controls. (B) Comparison of SF-36 results of patients with iTTP treated with caplacizumab and without caplacizumab (and healthy controls). (C) HADS anxiety and depression scores for healthy controls and patients with iTTP and for patients with iTTP treated with or without caplacizumab. HADS, Hospital Anxiety and Depression Scale; iTTP, immune-mediated thrombotic thrombocytopenic purpura; SF-36, Short Form-36.

Patients with iTTP had significantly lower scores in all domains in the SF-36 survey than healthy controls (*P* < .001 for all domains). Furthermore, MCS and PCS were significantly lower in patients with iTTP than those in the healthy controls (*P* < .001). No difference was observed between patients treated with caplacizumab and those without in the SF-36 survey (Table 2; Figure 2). Of note, age was a factor influencing significantly multiple domains of the SF-36 survey: advanced age was associated with improved general health perceptions, vitality, mental health, and MCS among patients with iTTP. Univariate and multivariate regression results for the SF-36 survey are detailed in Supplementary Tables 3–12.

**Table 2:** Results of the Short-Form-36 questionnaire assessing health-related quality of life.

Characteristics	iTTP (*n* = 101)	Healthy controls (*n* = 76)	*P*	iTTP, no caplacizumab (*n* = 56)	iTTP, caplacizumab (*n* = 45)	*P*
Physical functioning (%)	75 (55-95)	95 (85-100)	<0.001	78 (60-95)	75 (48-90)	.26
Role limitations due to physical problems (%)	50 (0-75)	100 (75-100)	<0.001	50 (6-100)	25 (25-75)	.26
Role limitations due to emotional problems (%)	67 (0-100)	100 (67-100)	<0.001	67 (67-100)	33 (33-100)	.37
Vitality (%)	45 (25-60)	65 (50-79)	<0.001	45 (25-65)	45 (20-60)	.59
Mental health (%)	56 (40-76)	80 (60-88)	<0.001	60 (44-76)	56 (40-76)	.97
Social functioning (%)	63 (50-88)	88 (50-100)	<0.001	63 (50-100)	63 (44-88)	.26
Bodily pain (%)	58 (45-90)	100 (70-100)	<0.001	68 (45-90)	55 (34-80)	.07
General health perceptions (%)	45 (33-65)	70 (60-90)	<0.001	48 (31-65)	40 (33-60)	.48
MCS	37 (25-50)	54 (40-59)	<0.001	39 (25-51)	32 (24-50)	.51
PCS	64 (60-66)	69 (66-72)	<0.001	64 (59-67)	63 (60-66)	.44

Values are median (IQR).

iTTP, immune-mediated thrombotic thrombocytopenic purpura; MCS, mental composite score; PCS, physical composite score.

### 3.3 Anxiety and depression

The HADS questionnaire showed significant differences between patients with iTTP and healthy controls. Patients with iTTP had a higher median anxiety score (*P* < .001) and higher depression scores (*P* < .001) than healthy controls (Table 3; Figure 2). More than half of patients with iTTP had at least mild anxiety (*n* = 61, 60%), while 40 patients (40%) had at least mild depression. Severe anxiety and depression were observed in 17% and 4% of patients with iTTP, respectively.

**Table 3:** Results of the Hospital Anxiety and Depression Scale.

Scores	iTTP (*n* = 101)	Healthy controls (*n* = 76)	*P*	iTTP, no caplacizumab (*n* = 56)	iTTP, caplacizumab (*n* = 45)	*P*
Anxiety score	9 (6-12)	6 (3-9)	<.001	9 (6-13)	10 (5-12)	.52
Score 0-7 (normal)	40	47		22	18	
Score 8-10 (mild)	20	16		11	9	
Score 11-14 (moderate)	24	9		10	14	
Score ≥15 (severe)	17	4		13	4	
Depression score	6 (3-10)	3 (1-5)	<.001	6 (2-9)	6 (4-10)	.23
Score 0-7 (normal)	61	64		37	24	
Score 8-10 (mild)	21	6		8	13	
Score 11-14 (moderate)	15	5		8	7	
Score ≥15 (severe)	4	1		3	1	

Values are median (IQR) or *n*.

iTTP, immune-mediated thrombotic thrombocytopenic purpura.

Patients treated with caplacizumab had anxiety and depression scores comparable with those of patients treated without caplacizumab (*P* = .54 and *P* = .23, respectively). However, severe anxiety was more prevalent in patients treated without caplacizumab than that in patients treated with caplacizumab (*P* = .048).

We next assessed possible risk factors for depression and anxiety in patients with iTTP using multivariate regression models. Advanced age was associated with lower anxiety scores, and previous ADAMTS-13 relapses led to lower anxiety scores. However, the duration of ADAMTS-13 activity of ≥20% before study was not associated with anxiety scores. Accordingly, severe anxiety was associated with younger age and no previous ADAMTS-13 relapses; furthermore, caplacizumab administration led to decreased risk of severe anxiety. Regarding depression scores, shorter time from diagnosis was associated with higher depression scores. Risk factors for severe depression could not have been calculated due to low event numbers. Detailed results of the regression models regarding the HADS questionnaires are added in Supplementary Tables 13–15.

### 3.4 Posttraumatic stress disease

The median PTSD screening score was 42 (IQR, 24-52); 46 patients (48%) had a PCL-S score of ≥43, suggestive of PTSD. There was no significant difference in PCL-S scores between patients treated with caplacizumab and those treated without. No significant risk factors for PTSD could be identified among age, sex, neurologic or cardiac involvement, number of TPE, clinical or ADAMTS-13 relapses, and length of intensive care unit hospitalization.

## 4 Discussion

Our findings present evidence of a significant deterioration in outcomes reflecting quality of life in iTTP survivors compared with the general French population, which is consistent with previous studies [4,7,21,22]. Patients with iTTP experience higher rates of anxiety, depression, more negative attitude to life, and low resilience than healthy controls, which are also observed in as severely debilitating diseases as sickle cell disease or in other critical illnesses including severe sepsis [[22], [23], [24], [25], [26], [27]]. A previous analysis in France assessing similar aspects of HRQoL involved iTTP and patients with hemolytic uremic syndrome with more severe disease presentation and intensive care unit hospitalization, while the patient population in our study consisted of well-characterized patients with iTTP, representing a broader spectrum of disease severity and thereby a more representative iTTP population [4].

We could identify factors impacting HRQoL and mental health. First, age seemed to be the most universal factor influencing both HRQoL and anxiety; patients with more advanced age reported more favorable outcomes, which could result from more resilience in older patients [28]. Second, the use of caplacizumab during the acute phase could be associated with less severe anxiety in iTTP survivors, possibly because of the decrease in the burden of care provided by caplacizumab [15]. Lastly, previous ADAMTS-13 relapses were associated with less severe anxiety, possibly following a growing familiarization of patients with their disease, with the finding that pre-emptive treatment is efficient in preventing clinical relapses [11,12].

In our study, the incidence of anxiety was higher in iTTP than that in the whole French population: based on our results, 60% of patients had signs of anxiety on the HADS questionnaire, while the estimated incidence of anxiety disorders in the general French population is 21.6% [29]. Of note, the incidence of anxiety was higher in our healthy control group than that previously reported in the general population, but our results might have been affected by the COVID-19 pandemic [29]. Similarly, 40% of patients with iTTP showed signs of depression in our study, compared with 14% in our healthy control population, which is comparable with the French general population [30,31].

The causes resulting in neurocognitive impairment still remain debated, while their understanding is key to better prevent their occurrence. A role for cerebral infarctions was suggested previously from a prospective study, where half of patients with iTTP in clinical remission had silent cerebral infarction assessed by magnetic resonance imaging, along with higher rates of cognitive impairment, including severe cognitive impairment [32]. In that regard, the association between reduced (though detectable) ADAMTS-13 levels during remission and ischemic stroke in iTTP survivors [33] suggests that a more stringent control of ADAMTS-13 activity could decrease the risk of ischemic stroke and thereby improve the cognitive status and HRQoL of patients with iTTP. Further investigations are urgently required to address more in detail this crucial aspect.

Our study has limitations. Selection bias might occur in case of autoreported questionnaires, as patients with higher symptom burden might be more motivated to response, whereas patients with severe depression or patients with severely decreased daily functioning might not respond. Educational level and French language skills might also have influence. Data were not collected on race or ethnicity from healthy controls and were not available for all patients with iTTP; therefore, it was not assessed in this analysis. Second, the questionnaires used in this study were validated as screening and not diagnostic tools, and we have no certainty that patients who screened positive would have fit an accurate diagnosis of PTSD or depression in an extensive psychiatric evaluation. Lastly, due to low event numbers, risk factors for severe depression could not been assessed. Despite these limitations, the signal from our analysis is clear in the sense that altered scores screening neurocognitive dysfunction, even if autoreported, should alarm practitioners and prompt further assessment by mental health professionals for an adapted support early in the management.

As the landscape of iTTP treatment is evolving, new therapeutic approaches might influence long-term HRQoL and neurocognitive impairment. Recent studies, including the phase 3 MAYARI trial, suggested that TPE treatment might be omitted from the standard acute care in iTTP for many patients, reducing the burden of care [34,35]. This most likely will have an important effect on long-term mental health of patients with iTTP.

In conclusion, we confirm from a large cohort of well-characterized population the high incidence of anxiety, depression, PTSD, and decreased HRQoL in iTTP survivors. Furthermore, we showed that caplacizumab is associated with decreased risk of severe anxiety. Our results highlight the importance of more systematic psychological care and support for patients with iTTP after the acute phase of the disease. Further works are now needed to better understand their cause and prevent their occurrence.

## References

[1] Joly B.S., Coppo P., Veyradier A. (2017). Thrombotic thrombocytopenic purpura. Blood.

[2] Kremer Hovinga J.A., Coppo P., Lämmle B., Moake J.L., Miyata T., Vanhoorelbeke K. (2017). Thrombotic thrombocytopenic purpura. Nat Rev Dis Primers.

[3] Coppo P., Bubenheim M., Benhamou Y., Völker L., Brinkkötter P., Kühne L. (2025). Caplacizumab use in immune-mediated thrombotic thrombocytopenic purpura: an international multicentre retrospective Cohort study (The Capla 1000+ project). EClinicalMedicine.

[4] Azoulay E., Souppart V., Kentish-Barnes N., Benhamou Y., Joly B.S., Zafrani L. (2023). Post-traumatic stress disorder and quality of life alterations in survivors of immune-mediated thrombotic thrombocytopenic purpura and atypical hemolytic and uremic syndrome. J Crit Care.

[5] Lewis Q.F., Lanneau M.S., Mathias S.D., Terrell D.R., Vesely S.K., George J.N. (2009). Long-term deficits in health-related quality of life after recovery from thrombotic thrombocytopenic purpura. Transfusion.

[6] Riva S., Mancini I., Maino A., Ferrari B., Artoni A., Agosti P. (2020). Long-term neuropsychological sequelae, emotional wellbeing and quality of life in patients with acquired thrombotic thrombocytopenic purpura. Haematologica.

[7] Cataland S.R., Scully M.A., Paskavitz J., Maruff P., Witkoff L., Jin M. (2011). Evidence of persistent neurologic injury following thrombotic thrombocytopenic purpura. Am J Hematol.

[8] Scully M., Cataland S.R., Peyvandi F., Coppo P., Knöbl P., Kremer Hovinga J.A. (2019). Caplacizumab treatment for acquired thrombotic thrombocytopenic purpura. N Engl J Med.

[9] Mulas O., Efficace F., Costa A., Baldi T., Zerbini F., Mantovani D. (2024). Long-term health-related quality of life and mental health in patients with immune thrombotic thrombocytopenic purpura. Ann Hematol.

[10] Zheng X.L., Vesely S.K., Cataland S.R., Coppo P., Geldziler B., Iorio A. (2020). ISTH guidelines for the diagnosis of thrombotic thrombocytopenic purpura. J Thromb Haemost.

[11] Hie M., Gay J., Galicier L., Provôt F., Presne C., Poullin P. (2014). Preemptive rituximab infusions after remission efficiently prevent relapses in acquired thrombotic thrombocytopenic purpura. Blood.

[12] Jestin M., Benhamou Y., Schelpe A.S., Roose E., Provôt F., Galicier L. (2018). Preemptive rituximab prevents long-term relapses in immune-mediated thrombotic thrombocytopenic purpura. Blood.

[13] Zheng X.L., Vesely S.K., Cataland S.R., Coppo P., Geldziler B., Iorio A. (2020). ISTH guidelines for treatment of thrombotic thrombocytopenic purpura. J Thromb Haemost.

[14] Cuker A., Cataland S.R., Coppo P., de la Rubia J., Friedman K.D., George J.N. (2021). Redefining outcomes in immune TTP: an international working group consensus report. Blood.

[15] Coppo P., Bubenheim M., Azoulay E., Galicier L., Malot S., Bigé N. (2021). A regimen with caplacizumab, immunosuppression, and plasma exchange prevents unfavorable outcomes in immune-mediated TTP. Blood.

[16] Froissart A., Buffet M., Veyradier A., Poullin P., Provôt F., Malot S. (2012). Efficacy and safety of first-line rituximab in severe, acquired thrombotic thrombocytopenic purpura with a suboptimal response to plasma exchange. Experience of the French Thrombotic Microangiopathies Reference Center. Crit Care Med.

[17] McHorney C.A., Ware J.E., Lu J.F., Sherbourne C.D. (1994). The MOS 36-item Short-Form Health Survey (SF-36): III. Tests of data quality, scaling assumptions, and reliability across diverse patient groups. Med Care.

[18] Leplège A., Ecosse E., Verdier A., Perneger T.V. (1998). The French SF-36 Health Survey: translation, cultural adaptation and preliminary psychometric evaluation. J Clin Epidemiol.

[19] Zigmond A.S., Snaith R.P. (1983). The hospital anxiety and depression scale. Acta Psychiatr Scand.

[20] DSM Library, Diagnostic and Statistical Manual of Mental Disorders Psychiatry Online. https://psychiatryonline.org/doi/book/10.1176/appi.books.9780890425596.

[21] Holmes S., Podger L., Bottomley C., Rzepa E., Bailey K.M.A., Chandler F. (2021). Survival after acute episodes of immune-mediated thrombotic thrombocytopenic purpura (iTTP)—cognitive functioning and health-related quality of life impact: a descriptive cross-sectional survey of adults living with iTTP in the United Kingdom. Hematology.

[22] Falter T., Böschen S., Schepers M., Beutel M., Lackner K., Scharrer I. (2021). Influence of personality, resilience and life conditions on depression and anxiety in 104 patients having survived acute autoimmune thrombotic thrombocytopenic purpura. J Clin Med.

[23] Oluwole O., Fertrin K.Y., Kruse-Jarres R. (2021). Neurocognitive assessment of adults with sickle cell disease: a descriptive study. Blood.

[24] Cooper O., McBain H., Tangayi S., Telfer P., Tsitsikas D., Yardumian A. (2019). Psychometric analysis of the adult sickle cell quality of life measurement information system (ACSQ-Me) in a UK population. Health Qual Life Outcomes.

[25] Dampier C., LeBeau P., Rhee S., Lieff S., Kesler K., Ballas S. (2011). Health-related quality of life in adults with sickle cell disease (SCD): a report from the comprehensive sickle cell centers clinical trial consortium. Am J Hematol.

[26] Goggin K.P., Lu L., Lee D.E., Howell C.R., Srivastava D., Brinkman T.M. (2024). Severe sepsis during treatment for childhood leukemia and sequelae among adult survivors. JAMA Netw Open.

[27] Winters B.D., Eberlein M., Leung J., Needham D.M., Pronovost P.J., Sevransky J.E. (2010). Long-term mortality and quality of life in sepsis: a systematic review. Crit Care Med.

[28] Terrill A.L., Molton I.R., Ehde D.M., Amtmann D., Bombardier C.H., Smith A.E. (2016). Resilience, age, and perceived symptoms in persons with long-term physical disabilities. J Health Psychol.

[29] Leray E., Camara A., Drapier D., Riou F., Bougeant N., Pelissolo A. (2011). Prevalence, characteristics and comorbidities of anxiety disorders in France: results from the “Mental Health in General Population” survey (MHGP). Eur Psychiatry.

[30] Fond G., Lancon C., Auquier P., Boyer L. (2019). [Prevalence of major depression in France in the general population and in specific populations from 2000 to 2018: a systematic review of the literature]. Presse Med.

[31] SPF Prévalence des épisodes dépressifs en France chez les 18-85 ans : résultats du Baromètre santé. https://www.santepubliquefrance.fr/import/prevalence-des-episodes-depressifs-en-france-chez-les-18-85-ans-resultats-du-barometre-sante-2021.

[32] Chaturvedi S., Yu J., Brown J., Wei A., Selvakumar S., Gerber G.F. (2023). Silent cerebral infarction during immune TTP remission: prevalence, predictors, and impact on cognition. Blood.

[33] Upreti H., Kasmani J., Dane K., Braunstein E.M., Streiff M.B., Shanbhag S. (2019). Reduced ADAMTS13 activity during TTP remission is associated with stroke in TTP survivors. Blood.

[34] Coppo P. (2025). Caplacizumab and immunosuppression without plasma exchange in thrombotic thrombocytopenic purpura. ISTH 2025 Congress; June.

[35] Völker L.A., Brinkkoetter P.T., Knöbl P.N., Krstic M., Kaufeld J., Menne J. (2020). Treatment of acquired thrombotic thrombocytopenic purpura without plasma exchange in selected patients under caplacizumab. J Thromb Haemost.

