# Supplementary material for: Health-related quality of life and mental health in autoimmune thrombotic thrombocytopenic purpura patients in the caplacizumab era
Source: Res Pract Thromb Haemost. 2025 Dec 9;10(1):103297. doi: 10.1016/j.rpth.2025.103297 (PMC12804100; doi:10.1016/j.rpth.2025.103297)
Supplement: Supplementary Material [file mmc1.docx]

**Supplementary data**

**Supplementary table 1.** Minimal sample size calculated by power analysis for the various multiple regression analyses.

|  | **Minimal sample size** |
| --- | --- |
| HADS anxiety score | 67 |
| HADS severe anxiety | 178 |
| HADS depression score | 76 |
| SF-36 role limitations due to physical problems | 40 |
| SF-36 vitality | 40 |
| SF-36 social functioning | 100 |
| SF-36 general health perceptions | 60 |
| SF-36 mental composite score | 76 |
| SF-36 physical composite score | 67 |

Abbreviations: HADS, Hospital Anxiety and Depression Scale; SF-36, Short-Form-36.

**Supplementary table 2.** Depression and anxiety (self-reported) in medical history in healthy controls and iTTP patients.

|  | **Healthy controls**  **N=76** | **iTTP patients**  **N=101** | **P-value** |
| --- | --- | --- | --- |
| Previous depression, n (%) | 16 (21%) | 29 (29%) | 0.23 |
| Previous anxiety, n (%) | 28 (37%) | 44 (44%) | 0.35 |
| Previous medical consultation for mood disorder or stress, n (%) | 24 (32%) | 31 (31%) | >0.999 |
| Previous medical consultation for mood disorder or insomnia, n (%) | 13 (17%) | 15 (15%) | 0.84 |

Categorial variables are provided as number (percentage). Abbreviations: iTTP, immune-mediated thrombotic thrombocytopenic purpura.

**Supplementary table 3.** Risk factors for SF-36 physical functioning by beta regression: results of the univariate regression models.

| **Physical functioning** | **Univariate analysis** | | |
| --- | --- | --- | --- |
| **Variable** | **OR** | **IC95%** | **P-value** |
| Sex | 0.72 | 0.44 ;1.18 | 0.19 |
| Age | 0.99 | 0.98 ;1.01 | 0.62 |
| Caplacizumab administration | 0.77 | 0.50 ;1.18 | 0.23 |
| Underlying comorbidities | 0.58 | 0.38 ;0.90 | 0.01 |
| Neurological involvement on MRI | 1.05 | 0.59 ;1.86 | 0.87 |
| Previous clinical relapse | 0.69 | 0.39 ;1.22 | 0.20 |
| Time from last clinical relapse (months) | 0.99 | 0.98 ;1.01 | 0.51 |
| Previous ADAMTS13 relapse | 1.18 | 0.75 ;1.85 | 0.47 |
| Time from last ADAMST13 relapse (months) | 0.99 | 0.97 ;1.01 | 0.27 |
| Duration of ADAMTS13 activity ≥ 20% before study (months) | 1.005 | 1.00 ;1.01 | 0.15 |
| Number of TPE | 1.01 | 0.98 ;1.03 | 0.62 |
| Treatment on ICU | 0.75 | 0.47 ;1.21 | 0.24 |
| Total duration of ICU hospitalization (days) | 0.98 | 0.95 ;1.01 | 0.16 |
| Follow-up since diagnosis (months) | 1.003 | 1.00 ;1.01 | 0.17 |

Abbreviations: ADAMTS13, A Disintegrin And Metalloproteinase with ThromboSpondin-1 motifs, 13th member; IC95%, 95% confidence interval; ICU, intensive care unit; MRI, magnetic resonance imaging; OR, odds ratio; SF-36, Short-Form-36; TPE, therapeutic plasma exchange. Statistically significant values appear in bold.

**Supplementary table 4.** Risk factors for SF-36 role limitations due to physical problems by beta regression: results of the uni- and multivariate regression models.

| **Role limitations due to physical problems** | **Univariate analysis** | | | **Multivariate analysis** | | |
| --- | --- | --- | --- | --- | --- | --- |
| **Variable** | **OR** | **IC95%** | **P-value** | **OR** | **IC95%** | **P-value** |
| Sex | 0.89 | 0.49 ;1.62 | 0.70 |  |  |  |
| Age | 1.01 | 0.99 ;1.03 | 0.29 |  |  |  |
| Caplacizumab administration | 0.76 | 0.45 ;1.29 | 0.31 |  |  |  |
| Underlying comorbidities | 0.81 | 0.48 ;1.37 | 0.43 |  |  |  |
| Neurological involvement on MRI | 0.88 | 0.44 ;1.75 | 0.71 |  |  |  |
| Previous clinical relapse | 1.06 | 0.53 ;2.12 | 0.87 |  |  |  |
| Time from last clinical relapse (months) | 1.003 | 0.99 ;1.02 | 0.64 |  |  |  |
| Previous ADAMTS13 relapse | 1.41 | 0.82 ;2.44 | 0.21 |  |  |  |
| Time from last ADAMST13 relapse (months) | 0.99 | 0.98 ;1.01 | 0.68 |  |  |  |
| Duration of ADAMTS13 activity ≥ 20% before study (months) | 1.005 | 1.00 ;1.01 | 0.25 |  |  |  |
| Number of TPE | 1.03 | 1.00 ;1.06 | 0.06 | 1.02 | 0.98 ;1.06 | 0.24 |
| Treatment on ICU | 1.12 | 0.63 ;1.99 | 0.69 |  |  |  |
| Total duration of ICU hospitalization (days) | 1.01 | 0.98 ;1.05 | 0.47 |  |  |  |
| Follow-up since diagnosis (months) | 1.006 | 1.00 ;1.01 | 0.06 | 1.003 | 0.99 ;1.01 | 0.32 |

Abbreviations: ADAMTS13, A Disintegrin And Metalloproteinase with ThromboSpondin-1 motifs, 13th member; IC95%, 95% confidence interval; ICU, intensive care unit; MRI, magnetic resonance imaging; OR, odds ratio; SF-36, Short-Form-36; TPE, therapeutic plasma exchange. Statistically significant values appear in bold.

**Supplementary table 5.** Risk factors for SF-36 role limitations due to emotional problems by beta regression: results of the univariate regression models.

| **Role limitations due to emotional problems** | **Univariate analysis** | | |
| --- | --- | --- | --- |
| **Variable** | **OR** | **IC95%** | **P-value** |
| Sex | 0.85 | 0.46 ;1.57 | 0.61 |
| Age | 1.003 | 0.99 ;1.02 | 0.71 |
| Caplacizumab administration | 0.81 | 0.48 ;1.38 | 0.45 |
| Underlying comorbidities | 0.75 | 0.44 ;1.29 | 0.29 |
| Neurological involvement on MRI | 0.77 | 0.38 ;1.57 | 0.47 |
| Previous clinical relapse | 1.17 | 0.58 ;2.4 | 0.67 |
| Time from last clinical relapse (months) | 0.99 | 0.98 ;1.01 | 0.47 |
| Previous ADAMTS13 relapse | 1.14 | 0.66 ;1.99 | 0.63 |
| Time from last ADAMST13 relapse (months) | 1.001 | 0.98 ;1.02 | 0.96 |
| Duration of ADAMTS13 activity ≥ 20% before study (months) | 1.005 | 1.00 ;1.01 | 0.24 |
| Number of TPE | 1.03 | 0.99 ;1.06 | 0.13 |
| Treatment on ICU | 1.03 | 0.57 ;1.84 | 0.93 |
| Total duration of ICU hospitalization (days) | 1.01 | 0.98 ;1.05 | 0.45 |
| Follow-up since diagnosis (months) | 1.004 | 1.00 ;1.01 | 0.16 |

Abbreviations: ADAMTS13, A Disintegrin And Metalloproteinase with ThromboSpondin-1 motifs, 13th member; IC95%, 95% confidence interval; ICU, intensive care unit; MRI, magnetic resonance imaging; OR, odds ratio; SF-36, Short-Form-36; TPE, therapeutic plasma exchange. Statistically significant values appear in bold.

**Supplementary table 6.** Risk factors for SF-36 vitality by beta regression: results of the uni- and multivariate regression models.

| **Vitality** | **Univariate analysis** | | | **Multivariate analysis** | | | |
| --- | --- | --- | --- | --- | --- | --- | --- |
| **Variable** | **OR** | **IC95%** | **P-value** | **OR** | **IC95%** | **P-value** |  |
| Sex | 1.10 | 0.71 ;1.69 | 0.67 |  |  |  |  |
| Age | 1.02 | 1.01 ;1.03 | **0.001** | 1.02 | 1.006 ;1.03 | **0.003** |  |
| Caplacizumab administration | 0.96 | 0.66 ;1.40 | 0.84 |  |  |  |  |
| Underlying comorbidities | 1.13 | 0.77 ;1.65 | 0.53 |  |  |  |  |
| Neurological involvement on MRI | 1.03 | 0.63 ;1.70 | 0.90 |  |  |  |  |
| Previous clinical relapse | 1.05 | 0.64 ;1.73 | 0.93 |  |  |  |  |
| Time from last clinical relapse (months) | 1.00 | 0.99 ;1.02 | 0.35 |  |  |  |  |
| Previous ADAMTS13 relapse | 0.93 | 0.63 ;1.38 | 0.73 |  |  |  |  |
| Time from last ADAMST13 relapse (months) | 1.001 | 0.99 ;1.01 | 0.55 |  |  |  |  |
| Duration of ADAMTS13 activity ≥ 20% before study (months) | 1.005 | 1.00 ;1.01 | 0.09 | 1.005 | 1.00 ;1.01 | 0.08 |  |
| Number of TPE | 1.005 | 0.98 ;1.03 | 0.66 |  |  |  |  |
| Treatment on ICU | 0.9 | 0.60 ;1.36 | 0.62 |  |  |  |  |
| Total duration of ICU hospitalization (days) | 0.10 | 0.97 ;1.02 | 0.80 |  |  |  |  |
| Follow-up since diagnosis (months) | 0.10 | 0.99 ;1.005 | 0.84 |  |  |  |  |

Abbreviations: ADAMTS13, A Disintegrin And Metalloproteinase with ThromboSpondin-1 motifs, 13th member; IC95%, 95% confidence interval; ICU, intensive care unit; MRI, magnetic resonance imaging; OR, odds ratio; SF-36, Short-Form-36; TPE, therapeutic plasma exchange. Statistically significant values appear in bold.

**Supplementary table 7.** Risk factors for SF-36 mental health by beta regression: results of the univariate regression models.

| **Mental health** | **Univariate analysis** | | |
| --- | --- | --- | --- |
| **Variable** | **OR** | **IC95%** | **P-value** |
| Sex | 0.93 | 0.63 ;1.37 | 0.72 |
| Age | 1.01 | 1.004 ;1.03 | 0.01 |
| Caplacizumab administration | 0.97 | 0.70 ;1.36 | 0.88 |
| Underlying comorbidities | 1.08 | 0.77 ;1.51 | 0.67 |
| Neurological involvement on MRI | 0.81 | 0.52 ;1.26 | 0.35 |
| Previous clinical relapse | 0.77 | 0.49 ;1.2 | 0.25 |
| Time from last clinical relapse (months) | 1.00 | 0.99 ; 1.01 | 0.85 |
| Previous ADAMTS13 relapse | 1.19 | 0.84 ;1.69 | 0.33 |
| Time from last ADAMST13 relapse (months) | 1.005 | 0.99 ;1.02 | 0.36 |
| Duration of ADAMTS13 activity ≥ 20% before study (months) | 1.002 | 1.00 ;1.01 | 0.53 |
| Number of TPE | 1.01 | 0.99 ;1.03 | 0.44 |
| Treatment on ICU | 0.78 | 0.54 ; 1.13 | 0.19 |
| Total duration of ICU hospitalization (days) | 0.28 | 0.97 ;1.01 | 0.28 |
| Follow-up since diagnosis (months) | 1.002 | 1.00 ;1.01 | 0.36 |

Abbreviations: ADAMTS13, A Disintegrin And Metalloproteinase with ThromboSpondin-1 motifs, 13th member; IC95%, 95% confidence interval; ICU, intensive care unit; MRI, magnetic resonance imaging; OR, odds ratio; SF-36, Short-Form-36; TPE, therapeutic plasma exchange. Statistically significant values appear in bold.

**Supplementary table 8.** Risk factors for SF-36 social functioning by beta regression: results of the uni- and multivariate regression models.

| **Social functioning** | **Univariate analysis** | | | | **Multivariate analysis** | | |
| --- | --- | --- | --- | --- | --- | --- | --- |
| **Variable** | **OR** | **IC95%** | **P-value** | **OR** | | **IC95%** | **P-value** |
| Sex | 1.02 | 0.61 ;1.70 | 0.95 |  | |  |  |
| Age | 1.02 | 1.01 ;1.04 | **0.003** | 0.98 | | 0.93 ;1.02 | 0.31 |
| Caplacizumab administration | 0.69 | 0.44 ;1.07 | 0.10 |  | |  |  |
| Underlying comorbidities | 1.46 | 0.93 ;2.30 | 1.00 |  | |  |  |
| Neurological involvement on MRI | 1.19 | 0.62 ;2.04 | 0.69 |  | |  |  |
| Previous clinical relapse | 0.66 | 0.36 ;1.20 | 0.16 |  | |  |  |
| Time since last clinical relapse (months) | 1.01 | 1 ;1.02 | 0.06 | 1.00 | | 0.97 ;1.02 | 0.88 |
| Previous ADAMTS13 relapse | 1.54 | 0.97 ;2.44 | 0.07 | 2.88 | | 0.85 ;9.77 | 0.09 |
| Time from last ADAMST13 relapse (months) | 0.99 | 0.98 ;1.01 | 0.53 |  | |  |  |
| Duration of ADAMTS13 activity ≥ 20% before study (months) | 1.00 | 1.00 ;1.01 | 0.27 |  | |  |  |
| Number of TPE | 1.03 | 1.00 ;1.05 | 0.07 | 1.12 | | 0.97 ;1.30 | 0.11 |
| Treatment on ICU | 0.67 | 0.41 ;1.10 | 0.11 |  | |  |  |
| Total duration of ICU hospitalization (days) | 1.01 | 0.98 ;1.04 | 0.56 |  | |  |  |
| Follow-up since diagnosis (months) | 1.00 | 1.00 ;1.01 | 0.06 | 1.00 | | 0.98 ;1.01 | 0.72 |

Abbreviations: ADAMTS13, A Disintegrin And Metalloproteinase with ThromboSpondin-1 motifs, 13th member; IC95%, 95% confidence interval; ICU, intensive care unit; MRI, magnetic resonance imaging; OR, odds ratio; SF-36, Short-Form-36; TPE, therapeutic plasma exchange. Statistically significant values appear in bold.

**Supplementary table 9.** Risk factors for SF-36 bodily pain by beta regression: results of the univariate regression models.

| **Bodily pain** | **Univariate analysis** | | |
| --- | --- | --- | --- |
| **Variable** | **OR** | **IC95%** | **P-value** |
| Sex | 0.94 | 0.55 ;1.59 | 0.81 |
| Age | 1.00 | 0.98 ;1.01 | 0.99 |
| Caplacizumab administration | 0.71 | 0.45 ;1.13 | 0.15 |
| Underlying comorbidities | 0.79 | 0.49 ;1.26 | 0.32 |
| Neurological involvement on MRI | 1.70 | 0.93 ;3.11 | 0.08 |
| Previous clinical relapse | 1.17 | 0.63 ;2.15 | 0.61 |
| Time from last clinical relapse (months) | 1.00 | 0.98 ;1.01 | 0.63 |
| Previous ADAMTS13 relapse | 1.31 | 0.81 ;2.11 | 0.27 |
| Time from last ADAMST13 relapse (months) | 0.99 | 0.98 ;1.01 | 0.34 |
| Duration of ADAMTS13 activity ≥ 20% before study (months) | 1.00 | 1.00 ;1.01 | 0.39 |
| Number of TPE | 1.01 | 0.98 ;1.04 | 0.38 |
| Treatment on ICU | 1.35 | 0.81 ;2.24 | 0.24 |
| Total duration of ICU hospitalization (days) | 1.01 | 0.98 ;1.04 | 0.54 |
| Follow-up since diagnosis (months) | 1.00 | 1.00 ;1.01 | 0.53 |

Abbreviations: ADAMTS13, A Disintegrin And Metalloproteinase with ThromboSpondin-1 motifs, 13th member; IC95%, 95% confidence interval; ICU, intensive care unit; MRI, magnetic resonance imaging; OR, odds ratio; SF-36, Short-Form-36; TPE, therapeutic plasma exchange. Statistically significant values appear in bold.

**Supplementary table 10.** Risk factors for SF-36 general health perceptions by beta regression: results of the uni- and multivariate regression models.

| **General health perceptions** | **Univariate analysis** | | | **Multivariate analysis** | | | |
| --- | --- | --- | --- | --- | --- | --- | --- |
| **Variable** | **OR** | **IC95%** | **P-value** | **OR** | **IC95%** | **P-value** |  |
| Sex | 0.91 | 0.60 ;1.37 | 0.65 |  |  |  |  |
| Age | 1.02 | 1.00 ;1.03 | **0.02** | 1.01 | 1.00 ;1.03 | **0.04** |  |
| Caplacizumab administration | 0.89 | 0.62 ;1.28 | 0.53 |  |  |  |  |
| Underlying comorbidities | 1.04 | 0.72 ;1.50 | 0.84 |  |  |  |  |
| Neurological involvement on MRI | 1.10 | 0.68 ;1.78 | 0.70 |  |  |  |  |
| Previous clinical relapse | 0.67 | 0.42 ;1.09 | 0.11 | 0.73 | 0.43 ;1.24 | 0.25 |  |
| Time from last clinical relapse (months) | 1.00 | 1.00 ;1.01 | 0.90 |  |  |  |  |
| Previous ADAMTS13 relapse | 1.03 | 0.71 ;1.50 | 0.88 |  |  |  |  |
| Time from last ADAMST13 relapse (months) | 1.00 | 0.99 ;1.01 | 0.68 |  |  |  |  |
| Duration of ADAMTS13 activity ≥ 20% before study (months) | 1.004 | 1.00 ; 1.01 | 0.16 |  |  |  |  |
| Number of TPE | 1.02 | 1.00 ;1.04 | 0.06 | 1.02 | 1.00 ;1.05 | 0.10 |  |
| Treatment on ICU | 0.93 | 0.63 ;1.38 | 0.73 |  |  |  |  |
| Total duration of ICU hospitalization (days) | 1.001 | 0.99 ;1.03 | 0.47 |  |  |  |  |
| Follow-up since diagnosis (months) | 1.002 | 1.00 ;1.01 | 0.43 |  |  |  |  |

Abbreviations: ADAMTS13, A Disintegrin And Metalloproteinase with ThromboSpondin-1 motifs, 13th member; IC95%, 95% confidence interval; ICU, intensive care unit; MRI, magnetic resonance imaging; OR, odds ratio; SF-36, Short-Form-36; TPE, therapeutic plasma exchange. Statistically significant values appear in bold.

**Supplementary table 11.** Risk factors for SF-36 mental composite score by linear regression: results of the uni- and multivariate regression models.

| **Mental composite score** | **Univariate analysis** | | | **Multivariate analysis** | | |
| --- | --- | --- | --- | --- | --- | --- |
| **Variable** | **β (coefficient)** | **IC95%** | **P-value** | **β (coefficient)** | **IC95%** | **P-value** |
| Sex | -1.57 | -8.92 ;5.78 | 0.67 |  |  |  |
| Age | 0.23 | 0.04 ;0.43 | **0.02** | 0.24 | 0.03 ;0.5 | **0.02** |
| Caplacizumab administration | -2.15 | -8.26 ;3.96 | 0.49 |  |  |  |
| Underlying comorbidities | 0.27 | -6.22 ;6.76 | 0.93 |  |  |  |
| Neurological involvement on MRI | -1.53 | -8.16 ;5.10 | 0.65 |  |  |  |
| Previous clinical relapse | -1.64 | -8.85 ;5.57 | 0.65 |  |  |  |
| Time from last clinical relapse (months) | -0.001 | -0.16 ;0.16 | 0.99 |  |  |  |
| Previous ADAMTS13 relapse | 4.12 | -2.17 ;10.41 | 0.20 |  |  |  |
| Time from last ADAMST13 relapse (months) | 0.05 | -0.12 ;0.22 | 0.55 |  |  |  |
| Duration of ADAMTS13 activity ≥ 20% before study (months) | 0.06 | -0.02 ;0.13 | 0.14 |  |  |  |
| Number of TPE | 0.28 | -0.05 ;0.61 | 0.09 | 0.09 | -0.26 ;0.45 | 0.61 |
| Treatment on ICU | -3.57 | -10.7 ;3.60 | 0.33 |  |  |  |
| Total duration of ICU hospitalization (days) | -0.02 | -0.39 ;0.34 | 0.89 |  |  |  |
| Follow-up since diagnosis (months) | 0.05 | -0.01 ;0.12 | 0.10 | 0.06 | -0.02 ;0.14 | 1.16 |

Abbreviations: ADAMTS13, A Disintegrin And Metalloproteinase with ThromboSpondin-1 motifs, 13th member; IC95%, 95% confidence interval; ICU, intensive care unit; MRI, magnetic resonance imaging; SF-36, Short-Form-36; TPE, therapeutic plasma exchange. Statistically significant values appear in bold.

**Supplementary table 12.** Risk factors for SF-36 physical composite score by linear regression: results of the uni- and multivariate regression models.

| **Physical composite score** | **Univariate analysis** | | | | **Multivariate analysis** | | | |
| --- | --- | --- | --- | --- | --- | --- | --- | --- |
| **Variable** | **β (coefficient)** | **IC95%** | **P-value** | **β (coefficient)** | | **IC95%** | **P-value** |  |
| Sex | -0.14 | -2.56 ;2.27 | 0.91 |  | |  |  |  |
| Age | 0.048 | -0.02 ;0.11 | 0.18 |  | |  |  |  |
| Caplacizumab administration | -0.84 | -3.01 ;1.32 | 0.44 |  | |  |  |  |
| Underlying comorbidities | -1.05 | -3.30 ;1.20 | 0.36 |  | |  |  |  |
| Neurological involvement on MRI | 2.27 | -1.67 ;6.20 | 0.26 |  | |  |  |  |
| Previous clinical relapse | -2.39 | -5.46 ;0.69 | 0.13 |  | |  |  |  |
| Time from last clinical relapse (months) | 0.003 | -0.45 ;0.05 | 0.91 |  | |  |  |  |
| Previous ADAMTS13 relapse | -0.88 | -3.1 ;1.34 | 0.43 |  | |  |  |  |
| Time from last ADAMST13 relapse (months) | -0.05 | -0.12 ;0.02 | 0.13 |  | |  |  |  |
| Duration of ADAMTS13 activity ≥ 20% before study (months) | 0.031 | 0.002 ;0.06 | **0.04** | 0.02 | | -0.006 ;0.05 | 0.11 |  |
| Number of TPE | 0.11 | -0.02 ;0.24 | 0.10 | 0.10 | | -0.11 ;0.32 | 0.34 |  |
| Treatment on ICU | -0.18 | -2.66 ;2.30 | 0.88 |  | |  |  |  |
| Total duration of ICU hospitalization (days) | 0.08 | -0.03 ;0.20 | 0.13 |  | |  |  |  |
| Follow-up since diagnosis (months) | 0.005 | -0.01 ;0.03 | 0.61 |  | |  |  |  |

Abbreviations: ADAMTS13, A Disintegrin And Metalloproteinase with ThromboSpondin-1 motifs, 13th member; IC95%, 95% confidence interval; ICU, intensive care unit; MRI, magnetic resonance imaging; SF-36, Short-Form-36; TPE, therapeutic plasma exchange. Statistically significant values appear in bold.

**Supplementary table 13.** Risk factors for HADS anxiety score by linear regression: results of the uni- and multivariate regression models.

| **Anxiety score** | **Univariate analysis** | | | **Multivariate analysis** | | |
| --- | --- | --- | --- | --- | --- | --- |
| **Variable** | **β (coefficient)** | **IC95%** | **P-value** | **β (coefficient)** | **IC95%** | **P-value** |
| Sex | 0.13 | -1.97 ;2.23 | 0.90 |  |  |  |
| Age | -0.11 | -.017 ;-0.05 | 0.0002 | -0.11 | -0.17 ;-0.06 | **0.0002** |
| Caplacizumab administration | -0.83 | -2.65 ;0.98 | 0.36 |  |  |  |
| Underlying comorbidities | -0.97 | -2.83 ;0.88 | 0.30 |  |  |  |
| Neurological involvement on MRI | 0.38 | -2.06 ;2.83 | 0.76 |  |  |  |
| Previous clinical relapse | 1.66 | -0.76 ;4.08 | 0.18 |  |  |  |
| Time from last clinical relapse (months) | 0.03 | -0.01 ;0.08 | 0.14 |  |  |  |
| Previous ADAMTS13 relapse | -1.74 | -3.62 ;0.15 | 0.070 | -1.92 | -3.68 ;-0.16 | **0.03** |
| Time from last ADAMST13 relapse (months) | -0.001 | -0.06 ;0.06 | 0.98 |  |  |  |
| Duration of ADAMTS13 activity ≥ 20% before study (months) | 0.01 | -0.01 ; 0.04 | 0.35 |  |  |  |
| Number of TPE | -0.1 | -0.14 ;0.09 | 0.63 |  |  |  |
| Treatment on ICU | -0.03 | -2.04 ;1.97 | 0.97 |  |  |  |
| Total duration of ICU hospitalization (days) | 0.07 | -0.04 ;0.18 | 0.23 |  |  |  |
| Follow-up since diagnosis (months) | -0.002 | -0.02 ;0.02 | 0.87 |  |  |  |

Abbreviations: ADAMTS13, A Disintegrin And Metalloproteinase with ThromboSpondin-1 motifs, 13th member; IC95%, 95% confidence interval; ICU, intensive care unit; MRI, magnetic resonance imaging; TPE, therapeutic plasma exchange. Statistically significant values appear in bold.

**Supplementary table 14.** Risk factors for HADS severe anxiety by logistic regression: results of the uni- and multivariate regression models.

| **Severe anxiety** | **Univariate analysis** | | | | **Multivariate analysis** | | | |
| --- | --- | --- | --- | --- | --- | --- | --- | --- |
| **Variable** | **OR** | **IC95%** | **P-value** | **OR** | | **IC95%** | **P-value** |  |
| Sex | 1.08 | 0.34 ;4.17 | 0.90 |  | |  |  |  |
| Age | 0.95 | 0.91 ;0.98 | 0.01 | 0.94 | | 0.89 ;0.98 | **0.01** |  |
| Caplacizumab administration | 0.32 | 0.08 ;1.00 | 0.06 | 0.20 | | 0.05 ;0.68 | **0.02** |  |
| Underlying comorbidities | 0.80 | 0.28 ;2.32 | 0.67 |  | |  |  |  |
| Neurological involvement on MRI | 1.04 | 0.22 ;3.74 | 0.95 |  | |  |  |  |
| Previous clinical relapse | 1.04 | 0.22 ;3.74 | 0.95 |  | |  |  |  |
| Time from last clinical relapse (months) | 1.02 | 1.00 ;1.05 | 0.11 |  | |  |  |  |
| Previous ADAMTS13 relapse | 0.32 | 0.07 ;1.07 | 0.09 | 0.18 | | 0.04 ;0.70 | **0.02** |  |
| Time from last ADAMST13 relapse (months) | 0.99 | 0.89 ;1.04 | 0.67 |  | |  |  |  |
| Duration of ADAMTS13 activity ≥ 20% before study (months) (log-transformed) | 1.75 | 1.01 ;3.45 | 0.07 |  | |  |  |  |
| Number of TPE | 1.01 | 0.94 ;1.07 | 0.78 |  | |  |  |  |
| Treatment on ICU | 1.38 | 0.44 ;5.26 | 0.60 |  | |  |  |  |
| Total duration of ICU hospitalization (days) (square-root transformed) | 1.49 | 1.01 ;2.27 | 0.05 |  | |  |  |  |
| Follow-up since diagnosis (months) | 1.001 | 0.99 ; 1.01 | 0.91 |  | |  |  |  |

Abbreviations: ADAMTS13, A Disintegrin And Metalloproteinase with ThromboSpondin-1 motifs, 13th member; IC95%, 95% confidence interval; ICU, intensive care unit; MRI, magnetic resonance imaging; OR, odds ratio; TPE, therapeutic plasma exchange. Statistically significant values appear in bold.

**Supplementary table 15.** Risk factors for HADS depression score by linear regression: results of the uni- and multivariate regression models.

| **Depression score** | **Univariate analysis** | | | | **Multivariate analysis** | | | |
| --- | --- | --- | --- | --- | --- | --- | --- | --- |
| **Variable** | **β (coefficient)** | **IC95%** | **P-value** | **β (coefficient)** | | **IC95%** | **P-value** |  |
| Sex | -0,82 | -3.03 ;1.37 | 0.46 |  | |  |  |  |
| Age | -0.03 | -0.10 ;0.03 | 0.32 |  | |  |  |  |
| Caplacizumab administration | 1.09 | -0.75 ;2.93 | 0.24 |  | |  |  |  |
| Underlying comorbidities | 0.49 | -1.42 ;2.41 | 0.61 |  | |  |  |  |
| Neurological involvement on MRI | 1.96 | -0.29 ;4.21 | 0.09 | 1.59 | | -0.7 ;3.89 | 0.17 |  |
| Previous clinical relapse | -1.05 | -3.21 ;1.10 | 0.33 |  | |  |  |  |
| Time from last clinical relapse (months) | -0.02 | -0.06 ;0.023 | 0.37 |  | |  |  |  |
| Previous ADAMTS13 relapse | -1.53 | -0.33 ;0.26 | 0.09 | -0.81 | | -2.77 ;1.14 | 0.41 |  |
| Time from last ADAMST13 relapse (months) | -0.002 | -0.04 ;0.04 | 0.91 |  | |  |  |  |
| Duration of ADAMTS13 activity ≥ 20% before study (months) | -0.02 | -0.04 ;0.01 | 0.14 |  | |  |  |  |
| Number of TPE | -0.07 | -0.20 ;0.06 | 0.28 |  | |  |  |  |
| Treatment on ICU | 0.12 | -1.90 ;2.14 | 0.91 |  | |  |  |  |
| Total duration of ICU hospitalization (days) | 0.03 | -0.11 ;0.17 | 0.64 |  | |  |  |  |
| Follow-up since diagnosis (months) | -0.03 | -0.04 ;-0.01 | **0.001** | -0.02 | | -0.042 ;-0.002 | **0.03** |  |

Abbreviations: ADAMTS13, A Disintegrin And Metalloproteinase with ThromboSpondin-1 motifs, 13th member; IC95%, 95% confidence interval; ICU, intensive care unit; MRI, magnetic resonance imaging; TPE, therapeutic plasma exchange. Statistically significant values appear in bold.
